# Comparison of 3D Gradient-Echo Versus 2D Sequences for Assessing Shoulder Joint Image Quality in MRI

**DOI:** 10.1155/2024/2244875

**Published:** 2024-10-11

**Authors:** Shapoor Shirani, Najmeh-Sadat Mousavi, Milad Ali Talib, Mohammad Ali Bagheri, Elahe Jazayeri Gharebagh, Qasim Abdulsahib Jaafar Hameed, Sadegh Dehghani

**Affiliations:** ^1^Tehran Heart Center, Tehran University of Medical Sciences, Tehran, Iran; ^2^Department of Radiology, College of Health and Medical Technologies, Al-Ayen University, Nasiriyah, Thi-Qar, Iraq; ^3^Radiation Sciences Department, School of Allied Medical Sciences, Tehran University of Medical Sciences, Tehran, Iran; ^4^Radiation Sciences Department, School of Allied Medical Sciences, International Campus, Tehran University of Medical Sciences, Tehran, Iran

**Keywords:** artifacts, DESS, image quality, magnetic resonance imaging, MEDIC, shoulder joint, three dimensional gradient-echo, TRUEFISP, VIBE

## Abstract

**Background:** Three-dimensional gradient-echo (3D-GRE) sequences provide isotropic or nearly isotropic 3D images, leading to better visualization of smaller structures, compared to two-dimensional (2D) sequences. The aim of this study was to prospectively compare 2D and 3D-GRE sequences in terms of key imaging metrics, including signal-to-noise ratio (SNR), contrast-to-noise ratio (CNR), glenohumeral joint space, image quality, artifacts, and acquisition time in shoulder joint images, using 1.5-T MRI scanner.

**Methods:** Thirty-five normal volunteers with no history of shoulder disorders prospectively underwent a shoulder MRI examination with conventional 2D sequences, including *T*_1_- and *T*_2_-weighted fast spin echo (T_1_/T_2_w FSE) as well as proton density-weighted FSE with fat saturation (PD-FS) followed by 3D-GRE sequences including VIBE, TRUEFISP, DESS, and MEDIC techniques. Two independent reviewers assessed all images of the shoulder joints. Pearson correlation coefficient and intra-RR were used for reliability test.

**Results:** Among 3D-GRE sequences, TRUEFISP showed significantly the best CNR between cartilage-bone (31.37 ± 2.57, *p* < 0.05) and cartilage-muscle (13.51 ± 1.14, *p* < 0.05). TRUEFISP also showed the highest SNR for cartilage (41.65 ± 2.19, *p* < 0.01) and muscle (26.71 ± 0.79, *p* < 0.05). Furthermore, 3D-GRE sequences showed significantly higher image quality, compared to 2D sequences (*p* < 0.001). Moreover, the acquisition time of the 3D-GRE sequences was considerably shorter than the total acquisition time of PD-FS sequences in three orientations (*p* < 0.01).

**Conclusions:** 3D-GRE sequences provide superior image quality and efficiency for evaluating articular joints, particularly in shoulder imaging. The TRUEFISP technique offers the best contrast and signal quality, making it a valuable tool in clinical practice.

## 1. Background

Magnetic resonance imaging (MRI) is widely used for shoulder joint evaluation due to its high spatial resolution and tissue contrast [1, 2]. While two-dimensional (2D) sequences like fast spin echo (FSE) provide a good spatial resolution, signal-to-noise ratio (SNR), and favorable contrast-to-noise ratio (CNR) in shoulder MRI, they suffer from partial-volume artifact (PVA) because of high slice thickness (about 3 mm). Furthermore, cartilage, ligaments, and tendons were oriented obliquely and difficult to evaluate with 2D sequences [1].

Alternatively, 3D high-resolution imaging offers improved visualization of anatomical structures from various angles, enhancing lesion detection rates and reducing acquisition times compared to 2D MRI in multiple plans (axial, sagittal oblique, coronal oblique) [3–5]. These techniques provide isotropic 3D images, allowing for multiplanar reconstruction (MPR) with different slice thicknesses [6–8]. Currently, 3D-FSE and 3D gradient echo (3D-GRE) sequences are used for MSK imaging.

3D-FSE sequences (e.g., 3D-FLAIR, 3D-T2, and 3D-SPACE) deliver submillimeter isotropic resolution while retaining the attributes of FSE sequences [9], but their drawback lies in lengthy scan times, typically around 10 min depending on parameters [2]. This impedes their routine clinical use. While 3D-GRE sequences generate high-resolution images with shorter acquisition time.

A variety of 3D-GRE sequences are available, including *T*_1_ volumetric interpolated breath-hold (VIBE), T_2_w true fast imaging with steady-state free precession (TRUFISP), T_2_w double-echo steady-state (DESS), and T_2_w multiecho data image combination (MEDIC) techniques [10, 11].

The VIBE is a GRE sequence with spoiler gradients added along the slice and readout directions to destroy the transverse signal. The TRUEFISP, a balanced steady-state free precession sequence, generates the high signal intensity (SI) from fluid, while the SI of the cartilage is preserved, leading to excellent contrast [12]. In the DESS sequence, two echoes are recorded and combined to obtain a higher *T*_2_^∗^ weighting with good contrast between cartilage and synovial fluid. This technique is characterized by a short repetition time (TR) which limits the decay of transverse magnetization before the next RF pulse is applied [13]. In MEDIC, multiple echoes are collected from a single excitation, resulting in reduced sensitivity to motion artifacts while improving the SNR of the image [14].

Several studies suggest that 3D sequences could improve the diagnostic capabilities of 2D techniques [2, 15, 16]. However, the role of GRE sequences is not clear, especially in joint imaging, and they may need to be combined with 2D sequences for lesion diagnosis. Therefore, further investigations are required to evaluate the performance and clinical efficacy of 3D techniques. Therefore, the aim of this study was to evaluate the performance of 3D-GRE sequences in terms of CNR, SNR, glenohumeral joint space, image quality, artifacts, and acquisition time in the assessment of shoulder joint, compared with 2D sequences.

## 2. Methods

Prior to the examination, all participants gave verbal and written informed consent to this study. This study was ethically approved by the local ethics committee.

### 2.1. Population

In this prospective comparative study, 35 normal adult volunteers with no history of shoulder disorders were scanned between March 2022 and October 2022. Volunteers with a shoulder that was too large for the shoulder coil, shoulder-related bone or soft tissue cancers, shoulder disorders (such as rotator cuff tears, labral injuries, or adhesive capsulitis), and general contraindications to MRI (such as claustrophobia, pacemakers, cochlear implants, or metal fragments in their body) were excluded.

### 2.2. MRI Exam

MRI exam was conducted using a 1.5-tesla (1.5-T) MRI scanner (MAGNETOM Area, Siemens Erlangen, Germany) with a 16-channel dedicated shoulder coil. Images were acquired of the shoulder joint positioned in the magnet's isocenter. To prevent movement, the shoulder joint was positioned well in the center of the coil and tightly fixed with cushions. All participants were examined with conventional 2D and 3D-GRE sequences during the same session randomly. 2D sequences included *T*_1_-weighted FSE (T_1_w FSE) in sagittal oblique orientation and T_2_w FSE in coronal oblique orientation and proton density-weighted FSE (PD-FS) in axial, coronal oblique, and sagittal oblique orientations. All 3D-GRE sequences were performed in the coronal oblique plane, including VIBE, TRUFISP, DESS, and MEDIC. A water-selective excitation pulse was used to suppress the fat in all 3D-GRE and PD-FS sequences. We optimized the 3D sequence parameters by adjusting various factors such as bandwidth, TE, TR, and flip angle to ensure consistency across all sequences and achieve acceptable image quality for each sequence.

Our objective was to compare the conventional 2D (T_2_w FSE and PD-FS in coronal oblique plane) with high resolution 3D-GRE sequences as efficiently as possible by using the same geometrical parameters and similar bandwidths (same chemical shifts) where possible. We used a 160 × 160 mm square field of view (FOV), the slice thickness of 0.8 mm for 3D-GRE and 3 mm for 2D sequences, and 256 × 256 acquisition matrix to cover almost the entire shoulder. The phase encoding direction was superior-inferior in the coronal plane. The bandwidth was set 220 Hz/pixel, corresponding to a chemical shift of 1 pixel at 1.5 T. Detailed scan parameters are listed in Table 1 for 3D-GRE and 2D sequences.

### 2.3. Image Analysis

Conventional 2D and 3D-GRE MRI images were independently analyzed by two radiologists, with 25 and 10 years of experience interpreting MSK MRI, and they were blinded to the type of images. Furthermore, every volunteer was analyzed twice by single observer with 1-month interval. Readers assessed the SNR, CNR, glenohumeral joint space, image quality, artifacts (motion, susceptibility, and magic angle artifacts), and acquisition time. When there was a lack of consensus between the two radiologists, a third radiologist with over 10 years of experience in MSK MRI was consulted to resolve any disagreements.

The CNR and SNR were calculated according to the following equations [16]: cartilage‐bone CNR = (cartilage SI − bone SI)/(standard deviation (SD) of background noise) and cartilage‐muscle CNR = (cartilage SI − muscle SI)/(SD of background noise).

The SNR of cartilage, bone, and muscle was calculated according to the following equations: cartilage SNR = (cartilage SI)/(SD of background noise), bone SNR = (bone SI)/(SD of background noise), and muscle SNR = (muscle SI)/(SD of background noise).

To calculate SNR of cartilage for each sequence, three regions of interests (ROIs) were manually drawn in the superior, middle, and inferior glenoid articular cartilage on one image of one sequence and then copied to the same image on other sequences. The SI of these ROIs were read by MicroDicom viewer software, and the mean of pixels' SI was assessed. For other tissues, three ROIs were drawn in the same place for all sequences and mean SI was calculated. Three other ROIs were located in different areas of the image background to measure the mean SD of background noise, and then, SNR and CNR were evaluated. The glenohumeral joint space was measured employing the distance tool of the software in the three different regions (superior, middle, and inferior) of the glenohumeral joint.

Both qualitative and quantitative analysis were used to evaluate image quality. Subjective image quality was assessed by using the following criteria: tissue contrast, edge sharpness, amount of noise, artifacts, and reformat quality. The quality score was based on ordinal five-point Likert-scale [17] and defined as (1) poor and unacceptable for diagnostic purposes, (2) adequate but poorer than average quality, (3) average quality of a diagnostic acceptable image, (4) above average quality, and (5) best quality.

### 2.4. Inter- and Intrarater Repeatability

In this study, two independent evaluations of all participants by a single observer were used to calculate intrarater repeatability, separated by 1 month, and it was expressed as an intraclass correlation coefficient (ICC). Furthermore, the interrater reliability coefficient in percent (IRR, %) was used to show the agreement among raters as a basis for our calculation [18]. As a first step, we calculated the number of ratings that are in agreement. In the second step, we determined how many ratings there are in total. After that, the percent agreement is calculated by dividing the number of agreement ratings by the number of ratings times 100.

### 2.5. Statistical Analysis

The statistical analysis was performed by Origin 2017 and SPSS 2022. Differences between the MR sequences were statistically significant if *p* < 0.05. The Pairwise Wilcoxon and Friedman tests were used for multiple testing to compare the performance and subjective image quality grading of all sequences. Categorical variables were evaluated using the chi-squared test. In cases where the analysis of variance test yielded significant results, post hoc tests were conducted to determine specific group differences. The Pearson correlation coefficient and intra-RR were employed for reliability testing.

## 3. Results

In this study, 14 females (mean age of 34.69 ± 14.49 years) and 21 males (mean age of 40.86 ± 16.36 years) were invited. Shoulder images were evaluated qualitatively and quantitatively, using six MRI sequences (VIBE, TRUFISP, DESS, MEDIC, T_2_w FSE, and PD-FS).

### 3.1. CNR Measurement

The mean CNR measured for cartilage-bone showed that the T_2_w FSE showed a CNR of 36.15 ± 3.69. TRUEFISP and MEDIC sequences demonstrated the CNR of 31.37 ± 2.57 and 24.79 ± 2.74, respectively, followed by VIBE (20.15 ± 1.72) and DESS (12.09 ± 0.93) sequences. T_2_w FSE and TRUEFISP showed significantly the best CNR between cartilage-bone compared to other sequences (*p* < 0.05). Additionally, PD-FS showed significantly the lowest CNR (6.42 ± 0.45) with *p* < 0.05. For cartilage-muscle contrast evaluation, the TRUEFISP sequence presented significantly the best CNR (13.51 ± 1.14, *p* < 0.05) followed by VIBE, MEDIC, and PD-FS sequences (5.48 ± 0.64, 7.12 ± 1.03, and 5.97 ± 0.73, respectively), whereas the DESS sequence showed the CNR of 4.34 ± 0.60.

However, TRUEFISP showed significantly the higher CNR for cartilage-bone and cartilage-muscle compared to 3D-GRE sequences (*p* < 0.05). The lowest CNR was achieved by PD-FS (6.42 ± 0.45) for cartilage-bone and T_2_w FSE (1.69 ± 0.27) for cartilage-muscle, compared to other sequences, with *p* < 0.05. The CNR measurements indicated very good agreement for inter- and intrarater reproducibility (IRR > 80% and ICC > 0.8) among all sequences. The detail of mean CNR of cartilage-bone and cartilage-muscle for 2D and 3D-GRE sequences are summarized in Table 2.

### 3.2. SNR Measurement

For SNR analysis of cartilage, TRUEFISP sequence showed significantly the best mean SNR (41.65 ± 2.19), compared to other sequences (*p* < 0.05), followed by VIBE, MEDIC, and PD-FS sequences (25.67 ± 1.44, 25.46 ± 1.89, and 19.89 ± 1.67, respectively). DESS sequence showed the SNR of 15.06 ± 1.26, and T_2_w FSE sequence showed clearly lowest SNR (6.49 ± 0.97, *p* < 0.05). In muscle, the best SNR was also shown for TRUEFISP sequence (26.71 ± 0.79) compared to other sequences (*p* < 0.05). The MEDIC and VIBE sequences presented the SNR of 21.09 ± 1.78 and 20.52 ± 1.40, respectively, followed by PD-FS and DESS sequences (13.90 ± 1.16 and 11.64 ± 1.28, respectively). The T_2_w FSE sequence showed significantly the lowest SNR (6.07 ± 0.72, *p* < 0.05). In addition, when we compared the mean SNR in different bone regions, the best SNR was achieved for T_2_w FSE sequence (42.79 ± 1.79), compared to other sequences (*p* < 0.05), followed by the PD-FS and TRUEFISP sequences (15.04 ± 0.62 and 11.44 ± 1.07, respectively). The VIBE, DESS, and MEDIC sequences showed the lower SNR (5.46 ± 0.89, 4.34 ± 0.61, and 4.02 ± 0.74, respectively). The SNR measurements showed very/good agreement for inter- and intrarater reproducibility (IRR > 80% and ICC > 0.8) among all sequences.  Table 3 summarizes the mean SNR details for cartilage, muscle, and bone using 2D and 3D-GRE sequences.

### 3.3. The Glenohumeral Joint Space Measurement

**Figure 1:**
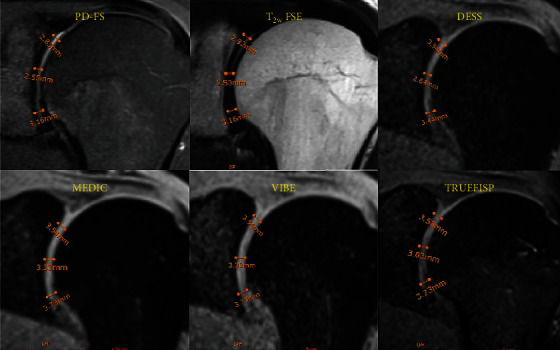
Conventional two-dimensional (2D) and three-dimensional (3D) MRI images of shoulder joint in a 51-year-old male. Glenohumeral space was measured in superior, middle, and inferior regions of the shoulder joint, and then, the mean glenohumeral space was calculated.

The glenohumeral joint space was measured at approximately superior, middle, and inferior regions of the shoulder joint (as shown in Figure 1), and efforts were made to ensure consistency in selecting the same regions for all volunteers. No significant differences were shown between 3D-GRE sequences (*p* > 0.05). However, the MEDIC sequence showed the mean articular space value (4.61 ± 0.77 mm) with a good agreement for inter- and intrarater reproducibility (IRR = 60.1% and ICC = 0.619). While TRUFISP indicated the mean articular space value (4.05 ± 0.66 mm) with a good agreement for inter- and intrarater reproducibility (IRR = 71.4% and ICC = 0.545). The mean articular space measured by VIBE and DESS was 4.50 ± 0.88 and 4.27 ± 0.78, respectively, with a good agreement for inter- and intrarater reproducibility (IRR = 69.4, ICC = 0.68 and IRR = 61.56, ICC = 0.73, respectively). The mean glenohumeral joint space was achieved 3.84 ± 0.52 mm for PD-FS (IRR = 51.1% and ICC = 0.548) and 4.45 ± 0.77 mm for T_2_w FSE (IRR = 11.63% and ICC = 0.674). The intrarater reproducibility measurement was achieved moderate for PD-FS and T_2_w FSE (≥ 0.50), while the interrater reproducibility measurement expressed moderate agreement for PD-FS (51.1%) and slight agreement for T_2_w FSE (11.63%). The details of the glenohumeral joint space measured in superior, middle, and inferior regions of the shoulder joint as well as the mean glenohumeral joint space are summarized in Table 4.

### 3.4. Image Quality Analysis

In terms of image quality, Likert scale was used to evaluate the MRI image quality. The number of occurance of a particular scale was measured for the 35 volunteers for all sequences. All 3D-GRE sequences more expressed an average image quality. Furthermore, all 3D-GRE sequences significantly showed higher image quality compared to T_2_w FSE and PD-FS sequences (*p* < 0.001). However, the number of occurrences of poor, adequate, average, above average, and best qualities for every observer is summarized in Table S1.

In 2D sequences, on the one hand, the T_2_w FSE sequence more exhibited adequate quality. On the other hand, the PD-FS sequence expressed all scale grades without any preferred grade or consistency in image quality.

**Figure 2:**
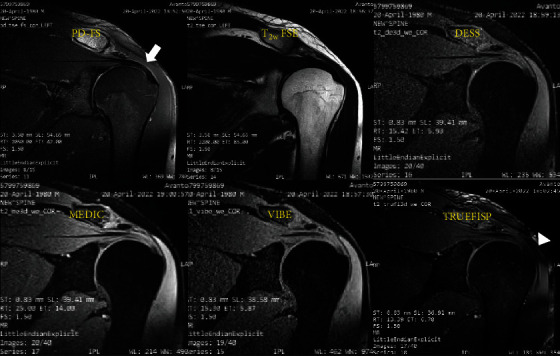
Conventional 2D and 3D MRI images of the right shoulder joint in a 44-year-old female. PD-FS image shows magic angle artifact (arrows). The TRUEFISP image reveals susceptibility artifact (arrowhead), whereas other 3D sequences show no artifacts.

After measuring artifacts, it was shown that the motion artifact was only present in T_2_w FSE and PD-FS sequences (20 and 7 out of 35, respectively), susceptibility artifacts were only exhibited in TRUFISP images (22 out of 35), and the magic angle artifact was only observed in PD-FS images (7 out of 35).  Figure 2 shows different types of artifacts obtained from a volunteer, using 2D and 3D sequences.

### 3.5. The Image Acquisition Time

The image acquisition time was 6 min 45 s for PD-FS in all planes (2 min 15 s for every plane; axial, coronal oblique, and sagittal oblique), 1 min 48 s for T_1_w FSE in sagittal oblique orientation, and 1 min 55 s for T_2_w FSE in coronal oblique plane. For 3D-GRE protocols, it took 3 min 17 s for VIBE, 3 min 15 s for TRUFISP, 3 min 40 s for DESS, and 3 min 30 s for MEDIC sequences. No significant differences were found between the acquisition times of 2D sequences (*p* > 0.05). 3D-GRE sequences also showed no significant differences (*p* > 0.05) in acquisition time, while the acquisition time for PD-FS in all planes was significantly higher than every 3D-GRE sequence (*p* < 0.01).

## 4. Discussion

This study is aimed at comparing the effectiveness of 3D-GRE sequences with routine 2D sequences in achieving optimal image quality. Current 2D sequences suffer from PVA due to thick sections (over 2 mm) and gaps between slices, limiting their ability to visualize small lesions [19, 20]. In contrast, 3D sequences offer isotropic imaging potential, allowing for MPR without sacrificing image quality, potentially replacing separately acquired 2D techniques, and saving time [21]. The results of this study indicate that 3D-GRE sequences, including MEDIC, VIBE, TRUEFISP, and DESS, offered good image quality. Among these sequences, TRUEFISP exhibited the best CNR for cartilage-bone and cartilage-muscle, as well as the highest SNR for cartilage and muscle, compared to other sequences.

The recent studies have also investigated the effectiveness of 3D-GRE sequences in evaluating articular joints [22, 23]. Commonly used clinical 3D-GRE-based methods, such as FLASH and DESS sequences, have been effective in assessing articular joints. However, other 3D sequences like VIBE, TRUFISP, SPACE, and MEDIC have also demonstrated success [24–26]. TRUFISP imaging, in particular, has shown excellent synovial fluid-cartilage contrast and utility in assessing various joint structures, including ligaments and cartilages [2, 27]. Furthermore, the use of high-field imaging at 3 T has been demonstrated to provide superior performance in articular joint imaging compared to imaging at 1.5 T. In the previous investigations, at 3 T, TRUEFISP was observed to provide excellent contrast between cartilage and joint fluid, with cartilage being visualized more effectively compared to SPACE sequences [9, 28, 29].

In this study, when comparing CNR between cartilage and surrounding tissues, T_2_w FSE and TRUEFISP showed significantly the best CNR between cartilage-bone compared to other sequences (*p* < 0.05). TRUEFISP also showed clearly the best cartilage-muscle CNR (*p* < 0.05) compared to other sequences. However, TRUEFISP showed significantly the higher CNR for cartilage-bone and cartilage-muscle compared to 3D-GRE sequences (*p* < 0.05). Additionally, DESS showed significantly lower CNR between cartilage-bone and cartilage-muscle compared to other 3D-GRE sequences. Notably, previous studies have suggested DESS as the best sequence for visualizing cartilage and synovial fluid, spatially in flip angles (FAs) close to 90° [15, 30]. But our results did not confirm them and the lowest CNR observed in DESS could be due to small FA (25°) used for this sequence. However, another study, on Knee MRI, reported the best cartilage-bone contrast for VIBE and SPACE sequences, followed by the DESS and MEDIC sequences with medium contrast which confirmed our work. But the TRUEFISP sequence showed the lowest contrast that was completely opposite to our results for this sequence [2]. However, difference in sequence parameters, magnetic field strength, and the type of tissue imaged could be the explainable reason for different results for the same sequence.

Furthermore, in our study, TRUEFISP also demonstrated significantly the higher mean SNR for cartilage and muscle (*p* < 0.05) compared to other sequences. These findings were consistent with the results reported by Mars et al. [2] and Abdulaal et al. [28], demonstrating the highest SNR for the TRUEFISP sequence compared to other sequences. Conversely, for the bone, T_2_w FSE indicated significantly higher SNR compared to other sequences, while TRUEFISP displayed superior SNR compared to other 3D-GRE sequences. Although 3D SPACE has been shown to be one of the best sequences for joint MRI, the relatively long acquisition time of this technique, compared to 3D-GRE sequences, is a major disadvantage for clinical imaging performed under economic constraints [16].

To detect the glenohumeral joint space, due to small cartilage thickness, we had to measure articular space to improve our accuracy in terms of glenohumeral joint space evaluation. In terms of glenohumeral joint space measurement, 3D-GRE sequences showed moderate reproducibility that could be due to high spatial resolution and excellent image quality, while 2D sequences displaying weak reproducibility results. The reasonable answer to slight agreement for T_2_w FSE could be the motion artifacts due to the heavy breathing of volunteers or the pulsation of the adjacent vessels which lead to lower image quality of this technique [9]. Additionally, the subsequent echoes in longer echo train lengths may lead to a reduction in overall SNR, CNR, and increased spatial blurring [7, 31].

3D sequences shed new light in MSK imaging. Despite longer acquisition times for isotropic 3D, compared to 2D sequences, they offer advantages such as MPRs and shorter total acquisition times when replacing multiple 2D acquisitions. Recent advancements in technology, such as parallel imaging and compressed sensing, have improved the clinical feasibility of 3D sequences [22, 32]. However, their adoption in standard clinical practice has been delayed due to longer acquisition times and sensitivity to patient motion, especially at 1.5 T [8, 33]. Nevertheless, as shown in this study, 3D sequences still offer shorter acquisition times compared to the cumulative time of multiple 2D PD-FS sequences in three planes [21].

The image quality of 3D-GRE sequences could be comparable with 2D sequences with higher image quality and lower PVA compared to 2D sequences [15, 16, 30]. In our study, all 3D sequences demonstrated significantly better image quality compared to 2D sequences (*p* < 0.05). Magic angle artifact is usually recognized in FSE sequences with TE ≤ 30 ms [34, 35], and our results confirmed that fact and all magic angle artifacts were shown in PD-FS. Furthermore, motion artifact was more seen in T_2_w FSE sequence followed by 2D PD-FS sequences. However, 3D acquisitions are more sensitive to subject motion, leading to potential corruption of the entire acquired k-space due to motion artifacts in patients without cooperation. This sensitivity arises from acquiring data over multiple slices or partitions in a single acquisition, where any motion during this process can introduce inconsistencies in the acquired data, ultimately impacting the final image quality [15].

It was shown that TRUEFISP is sensitive to field inhomogeneity, especially in higher magnetic fields. By employing strong volume shimming and utilizing a scout frequency for selecting the most appropriate frequency can effectively mitigate the sensitivity of TRUEFISP to field inhomogeneity [36]. Therefore, by performing shimming, for all 3D-GRE and PD-FS sequences, we were able to acquire images with good CNR, SNR, and average image quality for all sequences, confirming the findings of the previous study. Despite the susceptibility artifacts associated with TRUFISP due to its sensitivity to magnetic field inhomogeneities and frequency offsets, our shimming procedures allowed us to maintain good image quality for this sequence. However, it is important to note that these optimized parameters were designed for 1.5 T and may not perform optimally at higher field strengths. Additionally, in our observations, we noted that fat suppression appeared slightly compromised in the periphery of the shoulder near the skin border in both the TRUEFISP and PD-FS sequences. However, this subtle issue did not significantly affect the image quality of the shoulder joints in the scans.

### 4.1. Limitations

There are several limitations in our study. First, we did not compare the same type of pulse sequence in 2D and 3D approaches because of long acquisition time of 3D-FSE sequences (at least twice of 3D-GRE in our device). The second limitation of this study involved the relatively small number of normal volunteers which could be due to total long acquisition time (~30 min for both 2D and 3D sequences), leading to noncooperation of participants. Additionally, it is important to note that the performance of the different sequences in a clinical routine setting for patients with shoulder disorders was not evaluated in this study. Therefore, more studies are needed to evaluate the role of 3D sequences on diagnosis of different abnormalities in articular cartilage, glenoid labrum, biceps tendon, and rotator cuff in shoulder joints. The final limitation was the use of a 1.5 T MRI device instead of 3 T. High-field 3 T imaging is widely utilized in MSK imaging and, in most scenarios, has demonstrated superior image quality compared to 1.5 T.

## 5. Conclusions

3D-GRE sequences offered better image quality, compared to 2D sequences, making them suitable for articular joint assessment. Among 3D sequences, TRUEFISP exhibited significantly the best CNR between cartilage-bone and cartilage-muscle, as well as the highest SNR for cartilage and muscle, compared to other 3D sequences. Moreover, 3D sequences could take shorter acquisition time than the total acquisition time of all three 2D PD-FS sequences in three planes. Through further optimization, it is expected that 3D sequences reformatted into multiple planes will eventually be capable of replacing independently acquired 2D sequences, thereby significantly reducing the duration of shoulder magnetic resonance examinations.

## Figures and Tables

**Table 1:** Scanning parameters for three-dimensional (3D) and two-dimensional (2D) sequences used for assessment of the shoulder joint.

**Parameters**	**VIBE**	**TRUEFISP**	**DESS**	**MEDIC**	**T**_**2**_ **w FSE**	**PD-FS**
Repetition time (ms)	15.3	12.21	15.42	29	3500	3000
Echo time (ms)	5.87	4.12	5.93	14	80	20
Flip angle (°)	10	30	25	10	90	90
Bandwidth (Hz/pixel)	220	220	220	220	220	220
Slice oversampling (%)	50	50	50	50	50	50
Slice thickness (mm)	0.8	0.8	0.8	0.8	3	3
Acquisition time (min:s)	03:17	03:15	03:40	03:30	1:55	2:15
Acceleration factor (GRAPPA)	3	3	3	3	1.4	1.4
In-plane resolution	0.8 × 0.8	0.8 × 0.8	0.8 × 0.8	0.8 × 0.8	0.8 × 0.8	0.8 × 0.8
Fat suppression	WE	WE	WE	WE	—	WE
Volume shimming	Yes	Yes	Yes	Yes	No	Yes

Abbreviation: WE, water excitation.

**Table 2:** Summary of mean contrast-to-noise ratio (CNR) of cartilage-bone and cartilage-muscle and reader agreement across 2D and 3D-GRE sequences.

	**VIBE**	**TRUFISP**	**DESS**	**MEDIC**	**T**_**2**_ **w FSE**	**PD-FS**
Cartilage-bone	20.15 ± 1.72	31.37 ± 2.57	12.09 ± 0.93	24.79 ± 2.74	36.15 ± 3.69	6.42 ± 0.45
IRR (%)	94	96	91	86	84	89
ICC	0.87	0.87	0.84	0.80	0.80	0.80
Cartilage-muscle	5.48 ± 0.64	13.51 ± 1.14	4.34 ± 0.60	7.12 ± 1.03	1.69 ± 0.27	5.97 ± 0.73
IRR (%)	90	85	95	89	92	85
ICC	0.83	0.80	0.85	0.82	0.80	0.80

**Table 3:** Summary of mean signal-to-noise ratio (SNR) and reader agreement across different regions of cartilage, muscle, and bone.

	**VIBE**	**TRUFISP**	**DESS**	**MEDIC**	**T**_**2**_ **w FSE**	**PD-FS**
Cartilage						
Superior	25.40 ± 1.71	43.45 ± 2.53	13.72 ± 1.01	26.59 ± 2.12	5.92 ± 0.54	21.59 ± 1.14
Middle	24.39 ± 1.39	42.29 ± 3.31	15.25 ± 1.31	23.27 ± 1.10	7.62 ± 0.41	18.25 ± 2.41
Inferior	27.24 ± 2.48	39.21 ± 3.13	16.23 ± 1.63	26.52 ± 2.29	5.94 ± 0.49	19.85 ± 1.47
Mean	25.67 ± 1.44	41.65 ± 2.19	15.06 ± 1.26	25.46 ± 1.89	6.49 ± 0.97	19.89 ± 1.67
ICC	0.95	0.91	0.88	0.92	0.88	0.84
IRR (%)	87	86	82	87	82	80
Muscle						
Region 1	21.83 ± 2.13	26.81 ± 1.95	10.28 ± 1.05	19.84 ± 1.91	6.60 ± 0.38	12.93 ± 1.69
Region 2	20.72 ± 2.01	27.45 ± 1.94	12.84 ± 0.93	21.68 ± 1.90	5.25 ± 0.42	13.58 ± 1.72
Region 3	19.03 ± 2.59	25.87 ± 2.84	11.82 ± 1.27	21.75 ± 2.29	6.37 ± 0.59	15.19 ± 1.38
Mean	20.52 ± 1.40	26.71 ± 0.79	11.64 ± 1.28	21.09 ± 1.78	6.07 ± 0.72	13.90 ± 1.16
ICC	0.95	0.96	0.92	0.95	0.87	0.90
IRR (%)	89	90	88	91	82	85
Bone						
Region 1	6.37 ± 0.52	10.82 ± 0.94	5.02 ± 0.41	4.84 ± 0.38	43.28 ± 3.51	14.82 ± 1.37
Region 2	5.43 ± 0.49	12.68 ± 1.69	4.18 ± 0.39	3.84 ± 0.59	44.29 ± 3.40	14.86 ± 1.49
Region 3	4.59 ± 0.57	10.83 ± 1.40	3.83 ± 0.47	3.39 ± 0.63	40.81 ± 4.05	15.45 ± 2.51
Mean	5.46 ± 0.89	11.44 ± 1.07	4.34 ± 0.61	4.02 ± 0.74	42.79 ± 1.79	15.04 ± 0.62
ICC	0.91	0.96	0.94	0.90	0.87	0.84
IRR (%)	86	94	88	84	81	82

Abbreviations: ICC, intraclass correlation coefficient; IRR, interrater repeatability; Mean, mean value of the 3 ROIs.

**Table 4:** Summary of glenohumeral joint space measurements and reader agreement across, using 3D and 2D sequences.

**MRI sequence**	**Superior**	**Middle**	**Inferior**	**Mean (SD)**
VIBE	5.48 ± 0.72	3.75 ± 1.14	4.28 ± 0.64	4.50 ± 0.88
IRR	75.4%	74.8%	58.0%	69.4%
ICC	0.661	0.762	0.625	0.68
TRUFISP	4.75 ± 0.69	3.44 ± 0.56	3.96 ± 0.73	4.05 ± 0.66
IRR	70.4%	83.0%	60.8%	71.4%
ICC	0.553	0.715	0.369	0.545
DESS	5.11 ± 0.74	3.57 ± 0.80	4.13 ± 0.96	4.27 ± 0.78
IRR	72.2%	57.9%	54.6%	61.56%
ICC	0.760	0.760	0.627	0.730
MEDIC	5.48 ± 0.69	4.05 ± 0.48	4.30 ± 0.92	4.61 ± 0.77
IRR	70.6%	56.6%	53.1%	60.1%
ICC	0.573	0.805	0.479	0.619
T_2_w FSE	5.29 ± 0.61	3.78 ± 0.59	4.28 ± 1.15	4.45 ± 0.77
IRR	5.6%	8.4%	20.9%	11.63
ICC	0.705	0.720	0.599	0.674
PD-FS	4.44 ± 0.62	3.54 ± 0.48	3.54 ± 0.73	3.84 ± 0.52
IRR	44.3%	52.4%	56.6%	51.1%
ICC	0.599	0.672	0.374	0.548

Abbreviations: ICC, intraclass correlation coefficient; IRR, interrater repeatability; Mean, mean value of the three ROIs; SD, standard deviation.

## Data Availability

The datasets used and/or analyzed during the current study are available from the corresponding author on a reasonable request.

## Supporting Information

Supporting Information Additional supporting information can be found online in the Supporting Information section. Table S1. The number of occurrences of different image qualities according to five-point Likert-scale for every observer (S.H. and N.M.).
