# Supplementary material for: Comparison of 3D Gradient-Echo Versus 2D Sequences for Assessing Shoulder Joint Image Quality in MRI
Source: Int J Biomed Imaging. 2024 Oct 11;2024:2244875. doi: 10.1155/2024/2244875 (PMC11489005; doi:10.1155/2024/2244875)
Supplement: Supporting Information — Additional supporting information can be found online in the Supporting Information section. Table S1. The number of occurrences of different image qualities according to five-point Likert-scale for every observer (S.H. and N.M.). [file 2244875.f1.docx]

**Suppl. Table 1.** The number of occurrences of different image qualities according to 5-point Likert-scale for every observer (SH and NM).

|  |  | **Vibe** | **TRUFISD** | **DESS** | **Medic** | **T_2_ TSE** | **PD FS** |
| --- | --- | --- | --- | --- | --- | --- | --- |
| **SH (first reading)** | **Poor** | 0 | 1 | 2 | 0 | 7 | 4 |
|  | **Adequate** | 4 | 8 | 6 | 4 | 23 | 5 |
|  | **Average** | 24 | 17 | 22 | 20 | 3 | 8 |
|  | **Above average** | 7 | 6 | 4 | 10 | 1 | 7 |
|  | **Best** | 0 | 3 | 1 | 1 | 1 | 11 |
|  |  |  |  |  |  |  |  |
| **SH (second reading)** | **Poor** | 0 | 3 | 3 | 1 | 9 | 6 |
|  | **Adequate** | 8 | 6 | 4 | 5 | 18 | 6 |
|  | **Average** | 22 | 20 | 20 | 23 | 3 | 5 |
|  | **Above average** | 4 | 4 | 6 | 4 | 5 | 10 |
|  | **Best** | 1 | 2 | 2 | 2 |  | 8 |
|  |  |  |  |  |  |  |  |
| **NM (first reading)** | **Poor** | 2 | 2 | 4 | 2 | 5 | 9 |
|  | **Adequate** | 6 | 7 | 5 | 5 | 25 | 7 |
|  | **Average** | 20 | 19 | 19 | 23 | 4 | 8 |
|  | **Above average** | 5 | 6 | 7 | 5 | 1 | 7 |
|  | **Best** | 2 | 1 | 0 | 0 | 0 | 4 |
|  |  |  |  |  |  |  |  |
| **NM (second reading)** | **Poor** | 4 | 4 | 2 | 4 | 4 | 6 |
|  | **Adequate** | 5 | 5 | 7 | 6 | 20 | 9 |
|  | **Average** | 18 | 20 | 21 | 20 | 8 | 5 |
|  | **Above average** | 7 | 4 | 5 | 4 | 2 | 10 |
|  | **Best** | 1 | 2 | 0 | 1 | 1 | 5 |
